# Estimating SARS-CoV-2 infection probabilities with serological data and a Bayesian mixture model

**DOI:** 10.1038/s41598-024-60060-3

**Published:** 2024-04-25

**Authors:** Benjamin Glemain, Xavier de Lamballerie, Marie Zins, Gianluca Severi, Mathilde Touvier, Jean-François Deleuze, SAPRIS-SERO study group, Fabrice Carrat, Pierre-Yves Ancel, Marie-Aline Charles, Gianluca Severi, Mathilde Touvier, Marie Zins, Sofiane Kab, Adeline Renuy, Stephane Le-Got, Celine Ribet, Mireille Pellicer, Emmanuel Wiernik, Marcel Goldberg, Fanny Artaud, Pascale Gerbouin-Rérolle, Mélody Enguix, Camille Laplanche, Roselyn Gomes-Rima, Lyan Hoang, Emmanuelle Correia, Alpha Amadou Barry, Nadège Senina, Julien Allegre, Fabien Szabo de Edelenyi, Nathalie Druesne-Pecollo, Younes Esseddik, Serge Hercberg, Mélanie Deschasaux, Marie-Aline Charles, Valérie Benhammou, Anass Ritmi, Laetitia Marchand, Cecile Zaros, Elodie Lordmi, Adriana Candea, Sophie de Visme, Thierry Simeon, Xavier Thierry, Bertrand Geay, Marie-Noelle Dufourg, Karen Milcent, Delphine Rahib, Nathalie Lydie, Clovis Lusivika-Nzinga, Gregory Pannetier, Nathanael Lapidus, Isabelle Goderel, Céline Dorival, Jérôme Nicol, Olivier Robineau, Cindy Lai, Liza Belhadji, Hélène Esperou, Sandrine Couffin-Cadiergues, Jean-Marie Gagliolo, Hélène Blanché, Jean-Marc Sébaoun, Jean-Christophe Beaudoin, Laetitia Gressin, Valérie Morel, Ouissam Ouili, Jean-François Deleuze, Laetitia Ninove, Stéphane Priet, Paola Mariela Saba Villarroel, Toscane Fourié, Souand Mohamed Ali, Abdenour Amroun, Morgan Seston, Nazli Ayhan, Boris Pastorino, Xavier de Lamballerie, Nathanaël Lapidus, Fabrice Carrat

**Affiliations:** 1 Sorbonne Université, Inserm, Institut Pierre-Louis d’épidémiologie et de santé publique, Paris, France; 2 Département de santé publique, Hôpital Saint-Antoine, AP-HP. Sorbonne Université, Paris, France; 3 Unité des Virus Émergents, UVE, IRD 190, INSERM 1207, IHU Méditerranée Infection, Aix Marseille Univ, Marseille, France; 4 Paris University, Paris, France; 5 Université Paris-Saclay, Université de Paris, UVSQ, Inserm UMS 11, Villejuif, France; 6 CESP UMR1018, Université Paris-Saclay, UVSQ, Inserm, Gustave Roussy, Villejuif, France; 7 Department of Statistics, Computer Science and Applications, University of Florence, Florence, Italy; 8 Sorbonne Paris Nord University, Inserm U1153, Inrae U1125, Cnam, Nutritional Epidemiology Research Team (EREN), Epidemiology and Statistics Research Center, University of Paris (CRESS), Bobigny, France; 9 Fondation Jean Dausset-CEPH (Centre d’Etude du Polymorphisme Humain), CEPH-Biobank, Paris, France; 10 Centre for Research in Epidemiology and StatisticS (CRESS), Inserm, INRAE, Université de Paris, Paris, France; 11 EPIPAGE-2 Joint Unit, Paris, France; 12 ELFE Joint Unit, Paris, France; 13 Santé Publique France, Paris, France; 14 Inserm, Paris, France; 15 Aviesan, Inserm, Paris, France

**Keywords:** SARS-CoV-2, COVID-19, Bayes’ theorem, Mixture model, Epidemiology, Statistics, Statistical methods, Viral infection

## Abstract

The individual results of SARS-CoV-2 serological tests measured after the first pandemic wave of 2020 cannot be directly interpreted as a probability of having been infected. Plus, these results are usually returned as a binary or ternary variable, relying on predefined cut-offs. We propose a Bayesian mixture model to estimate individual infection probabilities, based on 81,797 continuous anti-spike IgG tests from Euroimmun collected in France after the first wave. This approach used serological results as a continuous variable, and was therefore not based on diagnostic cut-offs. Cumulative incidence, which is necessary to compute infection probabilities, was estimated according to age and administrative region. In France, we found that a “negative” or a “positive” test, as classified by the manufacturer, could correspond to a probability of infection as high as 61.8% or as low as 67.7%, respectively. “Indeterminate” tests encompassed probabilities of infection ranging from 10.8 to 96.6%. Our model estimated tailored individual probabilities of SARS-CoV-2 infection based on age, region, and serological result. It can be applied in other contexts, if estimates of cumulative incidence are available.

## Introduction

The first wave of the SARS-CoV-2 pandemic officially hit France on January 24, 2020, with the first European known cases at the time^1^. On March 17, a national lockdown was implemented for eight weeks, attenuating the virus circulation and its consequences in a non-immune population. The peak of COVID-19 related hospitalizations occurred during March-April, 2020^2^. Serological tests were performed at the end of this first wave to estimate the proportion of people who had been infected.

In France, several serosurveys were based on a test produced by Euroimmun to measure the presence of IgG targeting the S1 domain of the SARS-CoV-2 spike protein^3–5^. The results of this test are twofold. First, an ELISA ODR (optical density ratio) is returned, which is the ratio of the absorbance of the tested sample to the absorbance of a control sample. ELISA ODR is a continuous variable. Second, ELISA ODR is discretized into a ternary variable according to the manufacturer’s cut-offs: “negative” if ELISA ODR is below 0.8, “indeterminate” if ELISA ODR is between 0.8 and 1.1, and “positive” if ELISA ODR is above 1.1. Seroprevalence, the proportion of positive samples, was estimated to be about 5% in France and 10% in Ile-de-France (Paris area) using the 1.1 cut-off^3,5^. This 1.1 cut-off was associated with a sensitivity of 91.4% (92.7% when excluding tests realized less than 14 days after symptoms onset) and a specificity of 98.6%^6^. Sensitivity is notably limited by the presence of “non-responders”, an imperfectly-defined group of persons whose antibody levels do not increase, or increase only slightly, after the infection. The proportion of non-responders has been reported to be between 5 and 24%, depending on classification criteria and methodologies^7,8^.

Estimating cumulative incidence on the basis of serological data is done by correcting for the sensitivity and specificity of the serological test. This can be done through Bayesian methods, as a means of preserving uncertainty in the sensitivity and specificity estimates^9^. Other methodological challenges are linked to the selection of the analysis sample and it representativeness, and to potential biases in the selection of individuals in whom the sensitivity and specificity of the serological test are calculated with respect to the source population. A spectrum bias is indeed often suspected, as symptomatic individuals are more likely to be detected and therefore recruited to study sensitivity. These symptomatic persons are also more likely to have higher antibody levels^10–13^.

Mixture models constitute an appealing solution to spectrum bias. Indeed, the distribution of serological results is directly estimated from the sample of people whose infection status is unknown, which represents the target population. Hence, these models do not rely entirely on a possibly biased sample to estimate sensitivity^14,15^. In the case of serosurveys that took place after the first wave of COVID-19, a mixture model can be described as a weighted average of two probability distributions: one distribution for the serological results of infected persons, and one distribution for the serological results of uninfected persons. The weight which is associated to the infected persons is the cumulative incidence. These models are however prone to identification issues, corresponding to situations where more than one tuple of parameters’ values are consistent with the data. This situation happens notably when the two distributions overlap^14,16^.

Finally, estimating the probability of infection for a given individual can be enhanced by considering all the relevant information. First, the pre-test probability of infection in one individual corresponds to cumulative incidence. Thus, factors influencing cumulative incidence can be taken into account to modify this pre-test probability. Notably, it has been shown that seroprevalence varied significantly with administrative region and age class after the first wave in France^3^. Second, ELISA ODR, when considered as a continuous variable, varies within the categories of the discrete variable (“negative”, “indeterminate”, or “positive”). Hence, returning this ternary variable instead of the continuous ELISA ODR results in a loss of information. Indeed, modeling ELISA ODR as a continuous variable has been shown to outperform modeling it as a discrete variable in terms of bias and error^15^.

The main objective of this study was to propose a mixture model for estimating tailored individual infection probabilities after the first wave of SARS-CoV-2. To do so, we developed the model on French data, considering age and region, and modeling serological results as a continuous variable. We show the importance of not discretizing serological results for individual diagnosis. Our secondary objectives were to quantify the proportion of “non-responders”, and to estimate sensitivity and specificity for the serological test according to the manufacturer’s cut-offs. We also aimed to refine age-specific infection fatality rate and infection hospitalization rate using cumulative incidence estimates.

## Methods

### Serological data

The data of SAPRIS-SERO, a previously described serosurvey, were used in the present study^4,5,17^. SAPRIS-SERO is based on the SAPRIS cohort (“SAnté, Perception, pratiques, Relations et Inégalités Sociales en population générale pendant la crise COVID-19”), which was set up in March 2020 to study epidemiological and social features of the COVID-19 epidemic in France^17^. The adult participants of SAPRIS were recruited from three adult cohorts based on the general population:

- NutriNet-Santé is a general population cohort with online follow-up, focusing on nutrition. From the 170,000 participants included at the start of the study in 2009, 151,122 were still in the cohort in 2020^18^
- CONSTANCES is a general population cohort, set up in 2012, which includes 204,973 adults selected to be a representative sample of the French adult population^19^.
- E3N/E4N is a multi-generational adult cohort. It includes 113,000 persons: the women recruited at the start of the study (1990), their children, and the fathers of these children^20^.

All participants in these three initial cohorts with regular access to the Internet and still being followed in 2020 were invited to take part in the SAPRIS study, which consisted of self-administered questionnaires during the first wave. These questionnaires included notably demographic aspects and history of SARS-CoV-2 testing by RT-PCR. A total of 93,610 participants of SAPRIS were over 20, completed the questionnaires, and lived in metropolitan France. These participants were invited to take part in the SAPRIS-SERO study by taking a dried-blood spot by themselves. The samples were sent to a virology laboratory (Unité des virus émergents, Marseille, France) for serological analysis using the commercial ELISA test (Euroimmun, Lübeck, Germany) detecting anti-SARS-CoV-2 IgG directed against the S1 domain of the spike protein. The results of ELISA assays performed using dried-blood spot samples demonstrated a 98.1% to 100% sensitivity and a 99.3% to 100% specificity with conventional serum assays as a standard^21,22^. A maximum of one test per participant was performed, and an ELISA result was available for 82,467. Participants reporting a positive RT-PCR test were considered infected.

### Hospital and demographic data

The French population structure by 10-year age class and administrative region came from the Insee 2020 census (Institut national de la statistique et des études économiques)^23^. The data about COVID-19-related hospitalizations before the 1st of July 2020, by 10-year age class or by region, were obtained from SIVIC, the exhaustive national inpatient surveillance system used during the pandemic^24^. The data about general population mortality attributed to COVID-19 before the 1st of July 2020 were obtained from the CépiDc (Centre d’épidémiologie sur les causes médicales de décès)^25^

### Model

The statistical analysis was carried out within a Bayesian framework. In the rest of this section, prior distributions are not always explicitly written. If so, these distributions are uniform.

Serological results, originally expressed as optical density ratios (ODR), were modeled after a logarithmic transformation to be compatible with the use of unbounded probability functions. In the following, $P(\text {ELISA})$ refers to the distribution of log-ODR. *I* refers to the set of age classes (10-year groups, starting from 20 years, with persons over 90 included in the over 80 group), and *J* is the set of French administrative regions. The distribution of ELISA log-ODR in the persons whose infection status is unknown, considering an age class $i \in I$ and a region $j \in J$, was denoted $P(\text {ELISA} |i, j)$. This distribution was modeled as a mixture of the distributions $P(\text {ELISA}_+)$ and $P(\text {ELISA}_-)$, corresponding to the distributions of ELISA log-ODR in the infected and uninfected individuals, respectively. The proportion of persons having been infected during the first wave (cumulative incidence), given *i* and *j*, was written $p_{i, j}$:

$$\begin{aligned} P(\text {ELISA} |i, j) = p_{i, j} \times P(\text {ELISA}_{+}) + (1 - p_{i, j}) \times P(\text {ELISA}_{-}) \end{aligned}$$

In the uninfected individuals, ELISA log-ODR was modeled with a skew-normal distribution. The distribution of ELISA log-ODR in the infected individuals was itself a mixture of two normal distributions: one distribution for the responders, $P(\text {ELISA}_{\text {R}})$, and one distribution for the non-responders, $P(\text {ELISA}_{\text {NR}})$. The proportion of non-responders was written $p_{\text {NR}}$. A prior beta distribution for this proportion was specified to imply a prior 95% credible interval (95% CI) ranging from 1% to 40% (and thus covering the 5 to 24% estimates previously reported):^7,8^

$$\begin{aligned}&P(\text {ELISA}_{-}) = \text {Skew-normal}(\xi , \omega , \alpha )\\&P(\text {ELISA}_{+}) = (1 - p_{\text {NR}}) \times P(\text {ELISA}_\text {R}) + p_{\text {NR}} \times P(\text {ELISA}_{\text {NR}})\\&P(\text {ELISA}_{\text {R}}) = \text {Normal}(\mu _{\text {R}}, \sigma _{\text {R}})\\&P(\text {ELISA}_{\text {NR}}) = \text {Normal}(\mu _{\text {NR}}, \sigma _{\text {NR}})\\ \end{aligned}$$

Cumulative incidence on the logit scale, for an age class $i \in I$ and a region $j \in J$, was the sum of a regional intercept, $\alpha _j$, and of a log-odds ratio of age, $\beta _i$ without interaction:

$$\begin{aligned}&p_{i, j} = \frac{e^{y_{i, j}}}{1 + e^{y_{i, j}}}\\&y_{i, j} = \alpha _{j} + \beta _{i} \end{aligned}$$

A weakly informative normal prior distribution was specified for the age log-odds ratios ($\beta _{i}$), with mean 0 and standard deviation 1.

The cumulative distribution functions of ELISA log-ODR in the infected and uninfected individuals allowed estimating the sensitivity and specificity of the test as a binary variable, for several cut-offs. Using specificity and sensisivity, we estimated the area under the receiver operating curve (AUC), and the Younden’s J statistic. The Younden’s J statistic is the sum of specificity and sensitivity, minus one.

A potential decay of ELISA log-ODR over time was assessed with a frequentist linear regression in RT-PCR positive participants.

### Infection probability given ELISA ODR as continuous variable

The probability $p_{x, i, j}$ of having been infected given an ELISA ODR value *x*, an age group *i*, and a region *j*, was computed using Bayes’ rule. With $P( x | \text {infected})$ and $P(x | \text {uninfected})$ being the probability densities of the ELISA ODR value *x* in the infected and uninfected groups, respectively,

$$\begin{aligned} p_{x, i, j} = \frac{P(x | \text {infected}) \times p_{i, j}}{P(x | \text {infected}) \times p_{i, j} + P(x | \text {uninfected}) \times (1 - p_{i, j})} \end{aligned}$$

### Model comparison

We compared our model with alternative models using an approximation of the leave-one-out cross-validation using Pareto-smoothed importance sampling (PSIS-LOO)^26^. PSIS-LOO provides a Bayesian leave-one-out estimate of the expected log pointwise predictive density, with higher values indicating a better model for prediction. We used PSIS-LOO to assess the role of the non-responders component. We also compared the skew normal distribution of $P(\text {ELISA}_{-})$ with a normal distribution, and assessed the contribution of age and location to the fit.

### Post-stratified cumulative incidence and infection-outcome rates (external validity)

Cumulative incidence was reconstituted at the scale of age groups, regions, and at the scale of metropolitan France, to validate our model in the light of previous published studies. To correct for differences in age and geographical structures between the French population and the SAPRIS-SERO cohort, age-specific cumulative incidences were reconstructed by post-stratification from $p_{i, j}$ terms, considering the population size $\text {pop}_{i, j}$:

$$\begin{aligned} p_{\text {i}} = \sum _{j \in J} \frac{\text {pop}_{i, j}}{\text {pop}_{i}} \times p_{i, j} \end{aligned}$$

Region-specific cumulative incidence was computed according to the same method. Similarly, metropolitan France’s cumulative incidence was obtained from $p_i$ terms:

$$\begin{aligned} p_{\text {France}} = \sum _{i \in I} \frac{\text {pop}_{i}}{\text {pop}_{\text {France}}} \times p_{i} \end{aligned}$$

Infection hospitalization rate (IHR) and infection fatality rate (IFR) were also estimated, based on cumulative incidence, for comparison with previous studies. IHR and IFR correspond to the ratios of the number of hospitalizations or deaths attributed to COVID-19 to the number of infected persons.

### Algorithm and software

The data management used the R software version 4.2.3, and the modeling was done with the Stan software, which implements Hamiltonian Monte Carlo (R package cmdstanr version 2.32.0)^27,28^. The Monte Carlo sampling consisted in 6 chains of 2 000 iterations each (including 1 000 warm up iterations). Trace plots, $\hat{R}$ statistics and effective Monte Carlo sample sizes provided by Stan were used to assess convergence. Only two MCMC chains were run for PSIS-LOO estimation, due to memory usage. The model’s code (in Stan) is provided in Supplementary Code 1, and in a public repository available at https://github.com/bglemain/Refining-COVID-19-retrospective-diagnosis.

We encountered identification issues when fitting the mixture model, in the form of high $\hat{R}$ statistics, low effective sample sizes, and abnormal trace plots. We overcame these issues with a sequential approach. First, we estimated the distribution of ELISA ODR in infected individuals separately in a first model (319 persons with positive RT-PCR, see below). Second, we plugged the mean parameters’ estimates of this first model as data in the main model (this approach is called the plug-in principle)^29,30^. When computing sensitivity, AUC, and infection probability in the main model, uncertainty in the distribution of ELISA ODR in infected individuals was partially restored. This was done by drawing a set of parameters from a multi-normal approximation of the posterior distribution of the first model for each MCMC iteration of the main model.

### Ethical approval and consent to participate

Ethical approval and written or electronic informed consent were obtained from each participant before enrollment in the original cohort. The SAPRIS-SERO study was approved by the Sud-Mediterranée III ethics committee (approval 20.04.22.74247), and electronic informed consent was obtained from all participants for dried blood spot testing. The study was registered (#NCT04392388). All methods were performed in accordance with the relevant guidelines and regulations.

## Results

### Participants

All samples were collected between May and November 2020. Supplementary Figure 1 illustrates the timing of logical sampling and the timing of hospitalizations for COVID-19 in France during the first wave and early second wave. Among the total cohort of 82,467 participants with one serological test, 319 had a positive RT-PCR test. These 319 participants constituted the sample with known infection (mean age of 52 years, 29% men, mean elapsed time between the RT-PCR and dried blood sampling of 100 days, with a minimum of 12 days and a maximum of 190 days). After excluding 351 samples of individuals with missing data on administrative region of residence, the sample with participants of undetermined infection status included the remaining 81,797 participants (mean age 58 years, 35% men). No group of participants known uninfected was available. The number of observations for each region and each age group is provided in Supplementary Tables 5 and 6.

### Distribution of ELISA log-ODR

We did not find a significant decay of ELISA log-ODR over time in RT-PCR positive participants over the study period. The slope of the frequentist linear regression of ELISA log-ODR on the time between RT-PCR and serological testing was −0.03 (95% CI, −0.12 to 0.7, $p = 0.56$). Supplementary Figure 2 illustrates this result.

**Figure 1:**
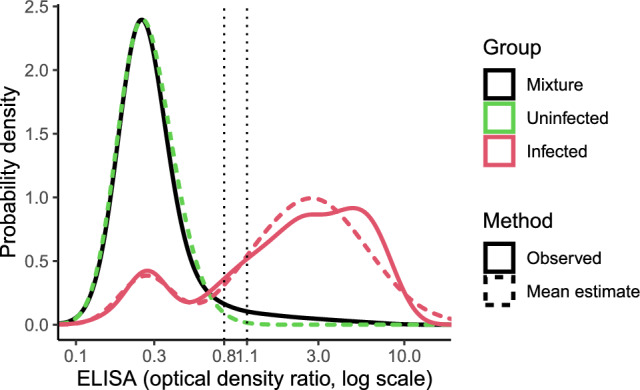
Observed and inferred ELISA ODR distributions.

The observed ELISA log-ODR distributions are displayed in Fig. 1, along with the distributions inferred by the model.

Among the infected individuals, the proportion of non-responders was estimated to be 14.5% (95% CI, 10.5–19.0%). The posterior estimates of the parameters involved in the distributions of ELISA log-ODR among the infected uninfected individuals are provided in Supplementary Tables 1-4.

These distributions imply an AUC of 92.3% (95% CI, 90.0% to 94.3%) for the serological test. Estimated sensitivities, specificities and Younden’s J statistics for the cut-offs 0.8 and 1.1 (ODR) are displayed in Table 1.

**Table 1:** Characteristics of the serological test depending on the cut-off.

	Cut-off: 0.8		Cut-off: 1.1	
Sensitivity (%)	81.1	$(77.2-85.0)$	75.9	$(71.7-80.0)$
Specificity (%)	99.8	$(99.7-99.8)$	100	$(99.9-100)$
J statistic*	80.9	$(77.0-84.8)$	75.9	$(71.7-80.0)$

^∗^Younden’s J statistic

### COVID-19 retrospective diagnosis: estimating individual infection probability

**Figure 2:**
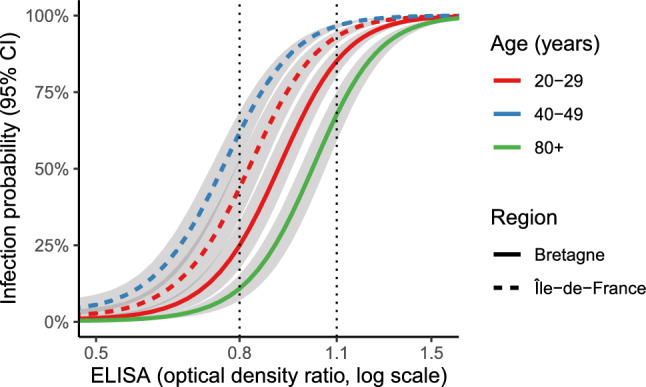
Influence of age, region, and ELISA ODR on the probability of infection.

The model was used to estimate infection probability at the individual scale in France, accounting for age, location (administrative region), and ELISA ODR as a continuous variable. Figure 2

illustrates how the probability of infection is related to ELISA ODR in two regions and three age groups representing the range of cumulative incidence. We found that a “negative” ELISA ODR (below 0.8) could be associated with an infection probability as high as 61.8% (95% CI, 52.7% to 68.6%), corresponding to an ELISA ODR of 0.8 for a person of 40-49 years living in Île-de-France (the region with the highest cumulative incidence). Conversely, a “positive” ELISA ODR (over 1.1) was compatible with a probability of infection as low as 67.7% (95% CI, 59.1% to 75.2%), corresponding to an ELISA ODR of 1.1 for a person over 80 living in Bretagne (the region with the lowest cumulative incidence). The “indeterminate” category (ODR from 0.8 to 1.1) encompassed highly variable probabilities of infection. Indeed, these probabilities ranged from 10.8% (95% CI%, 7.0% to 15.4%) for a person over 80 living in Bretagne and having an ELISA ODR of 0.8, to 96.6% (95% CI, 95.7% to 97.3%) for person of 40-49 years living in Île-de-France and having an ELISA ODR of 1.1. In this subsection, we did not consider the estimates of the region Corsica, due to the low count of tests made in this region. An exhaustive interactive table returning infection probability given age, region, and ELISA ODR is provided in the Supplementary media file.

### Model comparison

In the first model (distribution of ELISA log-ODR in the infected individuals), the PSIS-LOO estimate decreased from −437 to −449 when replacing the distribution of ELISA log-ODR in the infected individuals with a unique skew normal distribution. The PSIS-LOO estimate decreased from −437 to −478 when using a unique normal distribution.

In the main model, the PSIS-LOO estimate decreased from −39433 to −39605 when removing administrative region, from −39433 to −40255 when removing age, and from -39433 to -41779 when replacing the skew normal distribution of ELISA log-ODR in the uninfected individuals with a normal distribution.

### Cumulative incidence and infection-outcome rates

**Figure 3:**
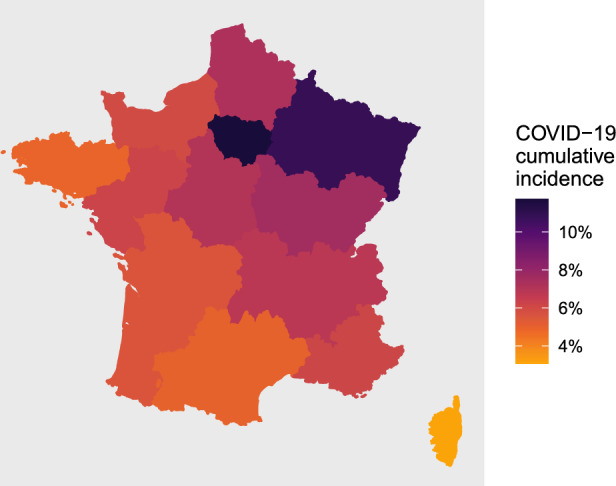
Regional cumulative incidence of COVID-19 after the first wave in metropolitan France. This map was created with the R packages maps version 3.4.1 (https://CRAN.R-project.org/package=maps) and ggplot2 version 3.4.4 (https://CRAN.R-project.org/package=ggplot2).

Cumulative incidence of COVID-19 among adults in metropolitan France after the first wave was 7.6% (95% CI, 7.3–7.8%), with a peak at 11.7% (95% CI, 11.1–12.4%) in Île-de-France (Paris area). Figure 3

features a map of metropolitan France showing the heterogeneity in cumulative incidence associated with location (exhaustive regional estimates are provided in Supplementary Table 7). IHR and IFR at the scale of metropolitan France were 2.6% (95% CI, 2.5% to 2.6%) and 0.8% (95% CI, 0.8% to 0.9%), respectively.

Age-specific cumulative incidence, IHR, and IFR, are presented in Table 2.

**Table 2:** Cumulative incidence and infection-outcome rates depending on age (mean estimates and 95% credible intervals).

Age	Cumulative incidence (%)		IHR (%)		IFR (%)	
$20-29$	7.1	$(5.8-8.6)$	0.4	$(0.3-0.5)$	0.01	$(0.00-0.01)$
$30-39$	13.6	$(12.7-14.4)$	0.4	$(0.4-0.4)$	0.01	$(0.01-0.01)$
$40-49$	13.5	$(12.8-14.1)$	0.6	$(0.6-0.6)$	0.03	$(0.02-0.03)$
$50-59$	5.8	$(5.3-6.2)$	2.5	$(2.3-2.7)$	0.2	$(0.2-0.2)$
$60-69$	3.4	$(3.1-3.7)$	6.4	$(5.8-7.1)$	1.0	$(0.9-1.1)$
$70-79$	3.1	$(2.8-3.3)$	11.6	$(10.6-12.6)$	3.2	$(2.9-3.5)$
$\ge 80$	2.6	$(2.0-3.2)$	33.7	$(26.3-43.4)$	21.6	$(16.9-27.8)$

IHR: infection hospitalization rate.

IFR: infection fatality rate

The two groups with the highest cumulative incidence were the 30-39 year-old persons (13.6%, 95% CI from 12.7% to 14.4%) and the 40-49 year-old persons (13.5%, 95% CI from 12.8% to 14.1%). IHR and IFR varied strongly with age, peaking respectively at 33.7% (95% CI from 26.2% to 13.3%) and 21.6% (95% CI from 16.8% to 27.8%) in persons older than 80 years.

## Discussion

We used a Bayesian mixture model to produce individual infection probability estimates in the context of the first wave of the SARS-CoV-2 pandemic in France. We showed that when considering age, region, and ELISA ODR as a continuous variable, each of the three categories of manufacturer’s classification covered a wide range of infection probabilities. Using the distributions of ELISA log-ODR inferred by the model, found a sensitivity of 75.9% for the 1.1 cut-off, which is below the 91.4% previously reported^6^. Specificity was high, even for the 0.8 cut-off (99.8%), in line with previous studies^6^. Among the infected individuals, the model estimated a proportion of non-responders of 14.5% (95% CI, 10.5-19.0%), in accordance with previous studies^7,8^

The model’s cumulative incidence estimates were in accordance with previously reported seroprevalence (about 5% in the whole country, and 10% in the most affected areas)^3–5,31^. Likewise, the highest cumulative incidence between 30 and 49 years that we found was in line with the higher seroprevalence previously reported in these age groups^3^. Infection hospitalization rate and infection fatality rate increased at exponential paces with age in adults, in a similar magnitude of those previously reported^31–35^

Other studies have sought to estimate the probability of SARS-CoV-2 infection, based on serological data in the form of a binary variable. These studies therefore estimated a positive predictive value and a negative predictive value. Based on the 1.1 cut-off, GeurtsvanKessel et al. (2020) showed that the same Euroimmun serological test as used in our study had a positive predictive value ranging from 84% to 100%, for a cumulative incidence ranging from 4% to 95%, respectively^36^. For the same interval of cumulative incidence, the test had a negative predictive value ranging from 22% to 99%. Using different serological tests and under varying prevalence, Brownstein and Chen (2021) showed that the proportion of positive tests being false ranged from 3% to 88%, while the proportion of negative tests being false remained below 10%^37^

Several studies have used mixture models in the context of the SARS-CoV-2 pandemic. Their objectives were to estimate cumulative incidence without relying on previously reported sensitivity and specificity, notably to correct for a possible spectrum bias. However, these studies did not use the model to generate individual-level probabilities of infection^14,15,38^. Bottomley et al. (2021) used a normal distribution for the uninfected individuals and a skew normal distribution for the infected individuals^14^. In the context of our data, we found that a skew normal distribution was more suitable to model the distribution of ELISA log-ODR in the uninfected individuals. The presence of the non-responders in the model improved the fit, as quantified by PSIS-LOO.

Several modeling assumptions were made. First, the distribution of ELISA log-ODR in the infected individuals did not take age into account. Similarly, the decrease of antibody levels with time was not modeled. Indeed, the waning of anti-spike 1 IgG was reported to be weak in the year after a natural SARS-CoV-2 infection, and the time between infection and testing could not exceed nine months in the current study^39^. When studying RT-PCR positive participants, we did not find a significant decrease in ELISA log-ODR over time.

Another limitation was due to identification issues, which are common in mixture models^16^. To overcome these identification issues, we estimated the distribution of ELISA log-ODR in the infected individuals based only on RT-PCR positive participants. Bottomley et al. (2021) used a similar approach, estimating some parameters in pre-COVID-19 samples and fixing these parameters afterward^14^. As a consequence, the uncertainty in cumulative incidence was under-estimated. This uncertainty was partially restored when computing sensitivity, AUC and infection probability. This sequential approach, known as the plug-in principle, has a second drawback. Indeed, a spectrum bias, if present, could not be taken into account as ELISA log-ODR distribution was only estimated from the RT-PCR positive participants.

Our method can also be used to calculate individual probabilities of infection after the first wave outside of France, given an ELISA ODR value and cumulative incidence estimates. An application based on published cumulative incidence estimates in New-York City and Connecticut is provided in the Supplementary information file.

In conclusion, the model estimated tailored individual infection probabilities based on age, region, and on a serological test modeled as a continuous variable.

### Supplementary Information

Supplementary Information 1.

Supplementary Information 2.

## Data Availability

The data of this study are under the protection of health data regulation, set by the French National Commission on Informatics and Liberty (Commission Nationale de l’Informatique et des Libertés, CNIL). The data can be made available upon reasonable request to fabrice.carrat@iplesp.upmc.fr, after a consultation with the steering committee of the SAPRIS-SERO study. The French law forbids us to provide free access to SAPRIS-SERO data; access could however be given by the steering committee after legal verification of the use of the data. Please, feel free to come back to us should you have any additional question.

## Code availability

The Stan code for the model is provided in the Supplementary information file, and in a public repository available at https://github.com/bglemain/Refining-COVID-19-retrospective-diagnosis.
