# Supplementary material for: Estimating SARS-CoV-2 infection probabilities with serological data and a Bayesian mixture model
Source: Sci Rep. 2024 Apr 25;14:9503. doi: 10.1038/s41598-024-60060-3 (PMC11045781; doi:10.1038/s41598-024-60060-3)
Supplement: Supplementary file 1 — Supplementary Information 1. [file 41598_2024_60060_MOESM1_ESM.pdf]

Estimating SARS-CoV-2 infection probabilities with serological data and a Bayesian mixture model

Supplementary information

Benjamin Glemain   Xavier de Lamballerie   Marie Zins
Gianluca Severi   Mathilde Touvier   Jean-François Deleuze
SAPRIS-SERO study group   Nathanaël Lapidus   Fabrice Carrat

Table of contents

Supplementary Figure 1: Timing of serological simpling in SAPRIS-SERO compared with COVID-19 related hospitalizations in France **3**

Supplementary Figure 2: ELISA ODR over time in RT-PCR positive participants **4**

Supplementary Tables 1-4: Parameters of the distribution of ELISA log-ODR **5**
Among the infected 5
Among the uninfected 5

Supplementary Table 5: Region of the participants **7**

Supplementary Table 6: Age of the participants **7**

Supplementary Table 7: Exhaustive regional cumulative incidence estimates **8**

Supplementary Code 1: Stan code **9**
Model of ELISA log-ODR in the infected 9
Main model (using the former's mean estimates) 9

Supplementary Code 2: Estimating individual infection probability outside of France given an ELISA ODR value and a cumulative incidence confidence interval **15**
Method 15
R code 15

Illustration in New-York city and Connecticut 19

References **20**

Supplementary Figure 1: Timing of serological simpling in SAPRIS-SERO compared with COVID-19 related hospitalizations in France

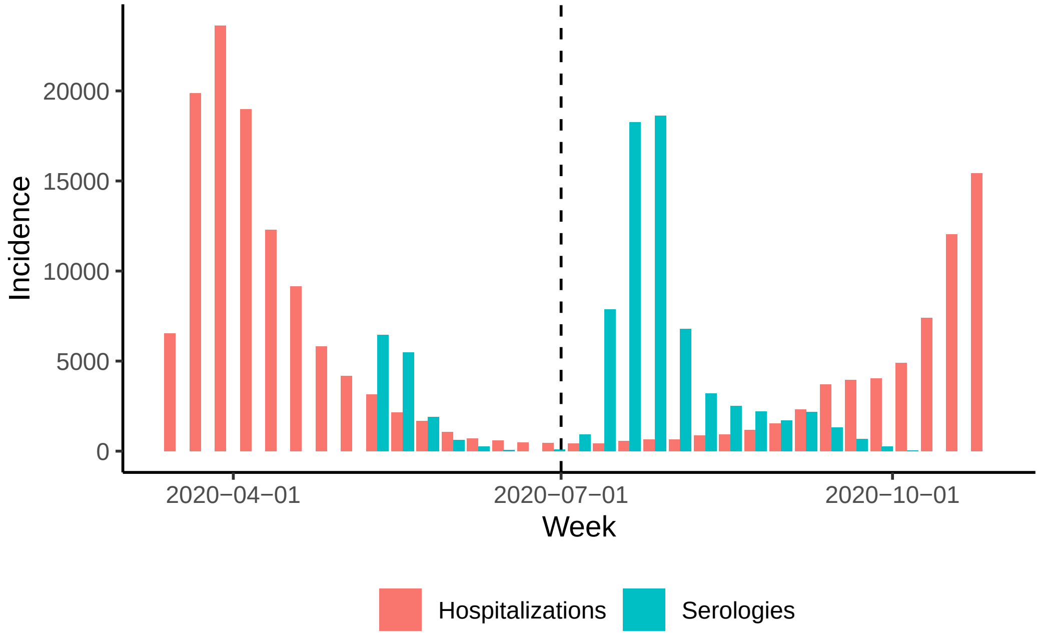

Figure 1: Weekly COVID-19 related hospitalizations in France and serological tests in SAPRIS-SERO

Supplementary Figure 2: ELISA ODR over time in RT-PCR positive participants

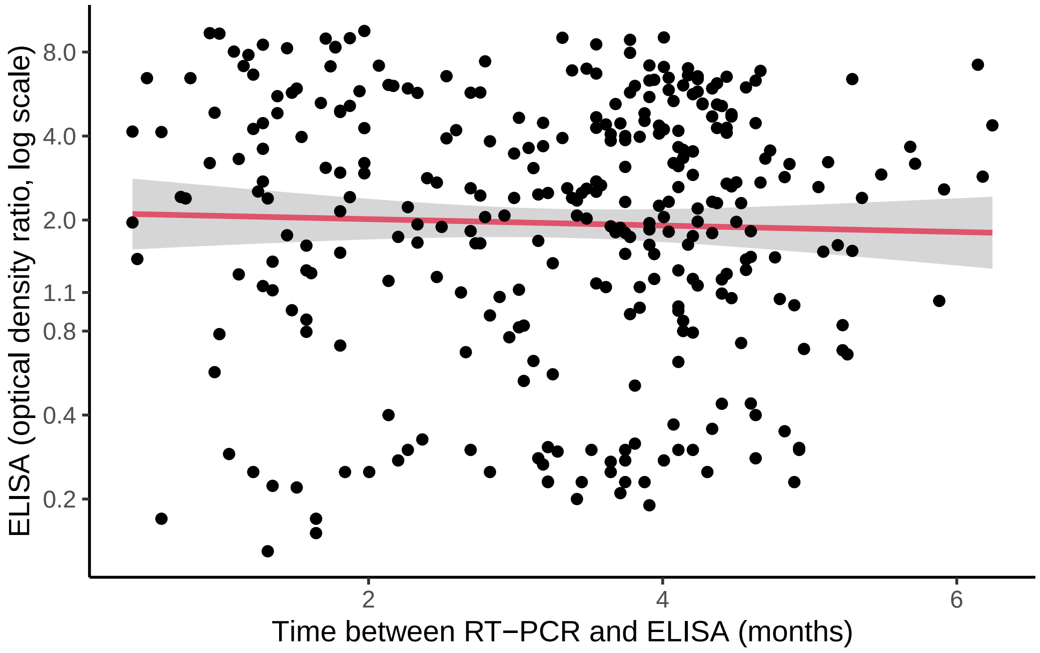

Figure 2: ELISA ODR (optical density ratio) over time in participants with a positive RT-PCR

Supplementary Tables 1-4: Parameters of the distribution of ELISA log-ODR

Among the infected

Below are the posterior mean and covariance matrix of the parameters of $P(\text{ELISA}_{+})$, the distribution of ELISA log-ODR in the infected:

Table 1: Mean posterior estimates of the distribution of ELISA log-ODR in the infected

	mean
mu_non_resp	-1.3271056
mu_resp	1.0050103
sigma_non_resp	0.2675957
sigma_resp	0.7482551
prop_non_resp	0.1448567

Table 2: Covariace matrix of the posterior of the distribution of ELISA log-ODR in the infected

	mu_non_resp	mu_resp	sigma_non_resp	sigma_resp	prop_non_resp
mu_non_resp	0.0023711	0.0003673	0.0007637	-0.0003349	0.0002114
mu_resp	0.0003673	0.0024387	0.0004025	-0.0003827	0.0001088
sigma_non_resp	0.0007637	0.0004025	0.0017769	-0.0003941	0.0002165
sigma_resp	-0.0003349	-0.0003827	-0.0003941	0.0015197	-0.0001508
prop_non_resp	0.0002114	0.0001088	0.0002165	-0.0001508	0.0004561

Among the uninfected

Below are the posterior mean and covariance matrix of the parameters of $P(\text{ELISA\_})$, the distribution of ELISA log-ODR in the uninfected:

Table 3: Mean posterior estimates of the distribution of ELISA log-ODR in the uninfected

	mean
xi_uninfected	-1.6621785
omega_uninfected	0.4745875
alpha_uninfected	2.5055857

Table 4: Covariace matrix of the posterior of the distribution of ELISA log-ODR in the uninfected

	xi_uninfected	omega_uninfected	alpha_uninfected
xi_uninfected	3.50e-06	-3.20e-06	-0.0000437
omega_uninfected	-3.20e-06	5.00e-06	0.0000506
alpha_uninfected	-4.37e-05	5.06e-05	0.0009138

Supplementary Table 5: Region of the participants

Table 5: Number of participants, by region

Region	N
Île-de-France	15444
Nouvelle-Aquitaine	10579
Auvergne-Rhône-Alpes	9526
Occitanie	8130
Grand Est	7146
Bretagne	5846
Provence-Alpes-Côte d'Azur	5015
Centre-Val de Loire	5010
Hauts-de-France	4892
Pays de la Loire	4598
Normandie	2769
Bourgogne-Franche-Comté	2757
Corse	85

Supplementary Table 6: Age of the participants

Table 6: Number of participants, by age class

Age	N
20-29	1574
30-39	8808
40-49	14681
50-59	14814
60-69	16642
70-79	22170
80+	3108

Supplementary Table 7: Exhaustive regional cumulative incidence estimates

Table 7: Cumulative incidence, by region

Region	Mean (%)	q2.5 (%)	q97.5 (%)	N
Île-de-France	11.7	11.1	12.4	15444
Grand Est	10.8	9.9	11.7	7146
Bourgogne-Franche-Comté	7.5	6.3	8.8	2757
Hauts-de-France	7.2	6.3	8.2	4892
Centre-Val de Loire	7.0	6.2	8.0	5010
Auvergne-Rhône-Alpes	6.8	6.1	7.4	9526
Pays de la Loire	6.2	5.4	7.1	4598
Provence-Alpes-Côte d'Azur	6.1	5.3	7.0	5015
Normandie	5.9	4.9	7.1	2769
Nouvelle-Aquitaine	5.6	5.0	6.2	10579
Occitanie	5.0	4.4	5.6	8130
Bretagne	4.9	4.2	5.6	5846
Corse	3.1	0.1	9.7	85

Supplementary Code 1: Stan code

Model of ELISA log-ODR in the infected

```
data {
  int<lower=0> N_pos;
  vector[N_pos] elisa_pos;
}


parameters {
  ordered[2] mu;
  vector<lower=0>[2] sigma;

  real<lower=0, upper=1> prop_non_resp;
}


model {

  // implies a prior 95\% CI ranging from 1 to 40\%
  prop_non_resp ~ beta(1.41, 8.6);

  for (n in 1:N_pos) {
    target += log_mix(
      prop_non_resp,
      normal_lpdf(elisa_pos[n] | mu[1], sigma[1]),
      normal_lpdf(elisa_pos[n] | mu[2], sigma[2])
    );
  }

}
```

Main model (using the former's mean estimates)

```
data {

  vector[5] mod1_params_mean;
  matrix[5, 5] mod1_params_Sigma;
```

```
  int<lower=0> N_mixt;
  vector[N_mixt] elisa_mixt;

  int<lower=0> N_region;
  array[N_mixt] int region;

  int<lower=0> N_age;
  array[N_mixt] int age;

  matrix<lower=0, upper=1>[N_age, N_region] mat_age_times_region;
  matrix<lower=0, upper=1>[N_region, N_age] mat_region_times_age;

  vector<lower=0, upper=1>[N_region] prop_region;
  vector<lower=0, upper=1>[N_age] prop_age;
  vector<lower=0, upper=1>[N_age] prop_hospit_age;
  real<lower=0> hospit_tot;
  vector<lower=0, upper=1>[N_age] prop_deces_age;
  real<lower=0> deces_tot;
  real<lower=0> pop_tot;

  int<lower=0> length_grid_sero;
  vector[length_grid_sero] grid_sero;

}


parameters {

  vector[N_age] log_or_age;
  vector[N_region] intercept_region;

  real xi_uninfected;
  real<lower=0> omega_uninfected;
  real alpha_uninfected;

}


transformed parameters {

  matrix<lower=0, upper=1>[N_age, N_region] incid_age_region;
```

```
  for (i in 1:N_age) {
    for (j in 1:N_region) {
      incid_age_region[i, j] = inv_logit(intercept_region[j] + log_or_age[i]);
    }
  }

}


model {

  log_or_age ~ normal(0, 1);

  for (n in 1:N_mixt) {
    target += log_mix(
      incid_age_region[age[n], region[n]],
      log_mix(
        mod1_params_mean[5],
        normal_lpdf(elisa_mixt[n] | mod1_params_mean[1], mod1_params_mean[3]),
        normal_lpdf(elisa_mixt[n] | mod1_params_mean[2], mod1_params_mean[4])
      ),
      skew_normal_lpdf(
        elisa_mixt[n] | xi_uninfected, omega_uninfected, alpha_uninfected
      )
    );
  }

}


generated quantities {

  // post-stratified incidences

  vector<lower=0, upper=1>[N_age] incid_age;
  for (i in 1:N_age) {
    incid_age[i] = incid_age_region[i, ] * mat_region_times_age[, i];
  }

  real<lower=0, upper=1> incid_fr_via_age = incid_age' * prop_age;
```

```
vector<lower=0, upper=1>[N_region] incid_region;
for (i in 1:N_region) {
  incid_region[i] = mat_age_times_region[, i]' * incid_age_region[, i];
}

real<lower=0, upper=1> incid_fr_via_region = incid_region' * prop_region;

// infection-outcome rates

vector<lower=0>[N_age] ihr_age;
for (i in 1:N_age) {
  ihr_age[i] = prop_hospit_age[i] / incid_age[i];
}

vector<lower=0>[N_age] ifr_age;
for (i in 1:N_age) {
  ifr_age[i] = prop_deces_age[i] / incid_age[i];
}

real<lower=0> ihr = hospit_tot / pop_tot / incid_fr_via_age;

real<lower=0> ifr = deces_tot / pop_tot / incid_fr_via_age;

// diagnostic performance of the serological test

vector[5] sample_params_mod1 = multi_normal_rng(
  mod1_params_mean, mod1_params_Sigma
);

real<lower=0, upper=1> sens08 = 1 -
    normal_cdf(log(.8) | sample_params_mod1[1], sample_params_mod1[3]) *
    sample_params_mod1[5] -
    normal_cdf(log(.8) | sample_params_mod1[2], sample_params_mod1[4]) *
    (1 - sample_params_mod1[5])
  ;

real<lower=0, upper=1> sens11 = 1 -
  normal_cdf(log(1.1) | sample_params_mod1[1], sample_params_mod1[3]) *
  sample_params_mod1[5] -
  normal_cdf(log(1.1) | sample_params_mod1[2], sample_params_mod1[4]) *
  (1 - sample_params_mod1[5])
```

```
;

real<lower=0, upper=1> spe08 = skew_normal_cdf(
  log(.8) | xi_uninfected, omega_uninfected, alpha_uninfected
);
real<lower=0, upper=1> spe11 = skew_normal_cdf(
  log(1.1) | xi_uninfected, omega_uninfected, alpha_uninfected
);

real<lower=0, upper=1> younden08 = sens08 + spe08 - 1;
real<lower=0, upper=1> younden11 = sens11 + spe11 - 1;

vector<lower=0, upper=1>[length_grid_sero] sens;
for (i in 1:length_grid_sero) {
  sens[i] = 1 -
    normal_cdf(grid_sero[i] | sample_params_mod1[1], sample_params_mod1[3]) *
    sample_params_mod1[5] -
    normal_cdf(grid_sero[i] | sample_params_mod1[2], sample_params_mod1[4]) *
    (1 - sample_params_mod1[5])
  ;
}

vector<lower=0, upper=1>[length_grid_sero] c1_spe;
for (i in 1:length_grid_sero) {
  c1_spe[i] = 1 - skew_normal_cdf(
    grid_sero[i] | xi_uninfected, omega_uninfected, alpha_uninfected
  );
}

real<lower=0, upper=1> AUC = (sens[1] + 1)/2 * (1 - c1_spe[1]);
for (i in 2:length_grid_sero) {
  AUC += (sens[i] + sens[i-1])/2 * (c1_spe[i-1] - c1_spe[i]);
}
AUC += sens[length_grid_sero]/2 * c1_spe[length_grid_sero];

// infection probability (given serology, age and region)

array[N_age, N_region, length_grid_sero] real pred_infection_age_region;
for (i in 1:N_age) {
  for (j in 1:N_region) {
    for (k in 1:length_grid_sero) {
    pred_infection_age_region[i, j, k] =
```

```
        exp(log_mix(
          sample_params_mod1[5],
          normal_lpdf(grid_sero[k] | sample_params_mod1[1], sample_params_mod1[3]),
          normal_lpdf(grid_sero[k] | sample_params_mod1[2], sample_params_mod1[4])
      )) * incid_age_region[i, j] /
        (
          exp(log_mix(
            sample_params_mod1[5],
            normal_lpdf(grid_sero[k] | sample_params_mod1[1], sample_params_mod1[3]),
            normal_lpdf(grid_sero[k] | sample_params_mod1[2], sample_params_mod1[4])
      )) * incid_age_region[i, j] +
          exp(skew_normal_lpdf(
            grid_sero[k] | xi_uninfected, omega_uninfected, alpha_uninfected)
          ) * (1 - incid_age_region[i, j])
        );
      }
    }
  }

  // log likelihood, to compute PSIS-LOO

  vector[N_mixt] log_lik;
  for (n in 1:N_mixt) {
    log_lik[n] = log_mix(
      incid_age_region[age[n], region[n]],
      log_mix(
        mod1_params_mean[5],
        normal_lpdf(elisa_mixt[n] | mod1_params_mean[1], mod1_params_mean[3]),
        normal_lpdf(elisa_mixt[n] | mod1_params_mean[2], mod1_params_mean[4])
      ),
      skew_normal_lpdf(
        elisa_mixt[n] | xi_uninfected, omega_uninfected, alpha_uninfected
      )
    );
  }

}
```

Supplementary Code 2: Estimating individual infection probability outside of France given an ELISA ODR value and a cumulative incidence confidence interval

Method

Individual infection probability can be estimated outside of France using (1) the posterior estimates of the distributions of ELISA log-ODR provided in Supplementary Tables 1-4, and (2) published estimates of cumulative incidence.
The procedure follows these steps:
1. Draw one set of parameters from a multivariate normal approximation of the distribution of ELISA log-ODR in the infected individuals
2. Likewise, draw one set of parameters for the uninfected individuals
3. Draw a cumulative incidence parameter in a beta distribution corresponding to the published 95% CI
4. Compute the probability of infection according to Bayes' theorem
5. Reiterate the four previous steps 1,000 times, compute the mean and the 95% CI using the quantiles

The R code below can be used to do this analysis. This code is also available at https://github.com/bglemain/Refining-COVID-19-retrospective-diagnosis.

R code

First, load the libraries, specify an ELISA ODR value (in this example: 1) and a 95% confidence interval for cumulative incidence (in this example: 20%-30%):

```
library(MASS)
library(tidyverse)
library(sn)
library(kableExtra)

elisa_odr <- 1 ## customize this value (ELISA ODR)

cuminc_low <- .2 ## customize this value
                 ## (lower limit of the 95% confidence
                 ## interval for cumulative incidence)

cuminc_high <- .3 ## customize this value
                  ## (higher limit of the 95% confidence
                  ## interval for cumulative incidence)
```

Then, find a beta distribution that matches the 95% CI and check it:

```
to_minimize <- function(param, ci_low, ci_high) {
  abs(
    qbeta(
      p = .025, shape1 = param[1], shape2 = param[2]
    ) - ci_low
  ) + abs(
    qbeta(
      p = .975, shape1 = param[1], shape2 = param[2]
    ) - ci_high
  )
}

init_cuminc <- list(alpha = 50, beta = 50)

optimized <- optim(
  par = init_cuminc, fn = to_minimize,
  ci_low = cuminc_low, ci_high = cuminc_high
)

## Checking that the beta distribution matches the 95% CI:
paste("CI low:", qbeta(
  p = .025,
  shape1 = optimized$par[["alpha"]],
  shape2 = optimized$par[["beta"]])
)
```

```
[1] "CI low: 0.199999966067442"
```

```
paste("CI high:", qbeta(
  p = .975,
  shape1 = optimized$par[["alpha"]],
  shape2 = optimized$par[["beta"]])
)
```

```
[1] "CI high: 0.299999983684483"
```

Then, store the posterior mean and covariance matrix of the distribution of ELISA log-ODR in the infected, and in the uninfected (provided in Supplementary Tables 1-4):

```r
## For the multivariate normal approximation of the
## distribution of ELISA log-ODR in the infected
mu_infected <- c(
  mu_non_resp = -1.3271056,
  mu_resp = 1.0050103,
  sigma_non_resp = 0.2675957,
  sigma_resp = 0.7482551,
  prop_non_resp = 0.1448567
)

Sigma_infected <- tribble(
  ~mu_non_resp, ~mu_resp,   ~sigma_non_resp,  ~sigma_resp,  ~prop_non_resp,
  0.00237,      0.000367,     0.000764,    -0.000335,        0.000211,
  0.000367,     0.00244,      0.000402,    -0.000383,        0.000109,
  0.000764,     0.000402,     0.00178,     -0.000394,        0.000217,
  -0.000335,   -0.000383,    -0.000394,     0.00152,        -0.000151,
  0.000211,     0.000109,     0.000217,    -0.000151,        0.000456
) |>
  as.matrix()

## For the multivariate normal approximation of the
## distribution of ELISA log-ODR in the uninfected
mu_uninfected <- c(
  xi_uninf = -1.6621785,
  omega_uninf = 0.4745875,
  alpha_uninf = 2.5055857
)

Sigma_uninfected <- tribble(
  ~xi_uninf,          ~omega_uninf,     ~alpha_uninf,
  3.514406e-06,      -3.187074e-06,    -4.373939e-05,
  -3.187074e-06,      5.042344e-06,     5.060927e-05,
  -4.373939e-05,      5.060927e-05,     9.138356e-04
) |>
  as.matrix()
```

Then, create a function to draw a set of parameters (from a multivariate normal distribution) and to compute the infection probability accordingly:

```r
draw_prob_infected <- function(
    elisa_odr, cuminc_alpha, cuminc_beta,
```

```
    mu_infected, Sigma_infected,
    mu_uninfected, Sigma_uninfected
) {

  cuminc <- rbeta(n = 1, shape1 = cuminc_alpha, shape2 = cuminc_beta)
  params_infected <- mvrnorm(n = 1, mu = mu_infected, Sigma = Sigma_infected)
  params_uninfected <- mvrnorm(n = 1, mu = mu_uninfected, Sigma = Sigma_uninfected)

  log_odr <- log(elisa_odr)

  lik_infected <-
    dnorm(
      log_odr,
      params_infected[["mu_non_resp"]],
      params_infected[["sigma_non_resp"]]
    ) *
    params_infected[["prop_non_resp"]] +
    dnorm(
      log_odr,
      params_infected[["mu_resp"]],
      params_infected[["sigma_resp"]]
    ) *
    (1 - params_infected[["prop_non_resp"]])

  lik_uninfected <- dsn(
    log_odr,
    xi = params_uninfected[["xi_uninf"]],
    omega = params_uninfected[["omega_uninf"]],
    alpha = params_uninfected[["alpha_uninf"]]
  )

  tibble(
    elisa_odr = elisa_odr,
    prob_infected = lik_infected * cuminc /
    (lik_infected * cuminc + lik_uninfected * (1 - cuminc))
  )

}
```

Finally, compute the infection probability for 1,000 sets of parameters, and compute the mean posterior and 95% CI from these values:

```r
draws_prob_infected <- map_dfr(
  1:1e3,
  ~ draw_prob_infected(
    elisa_odr = elisa_odr,
    cuminc_alpha = optimized$par[["alpha"]], cuminc_beta = optimized$par[["beta"]],
    mu_infected = mu_infected, Sigma_infected = Sigma_infected,
    mu_uninfected = mu_uninfected, Sigma_uninfected = Sigma_uninfected
  )
)

draws_prob_infected |>
  summarise(
    mean = mean(prob_infected),
    q2.5 = quantile(prob_infected, .025),
    q97.5 = quantile(prob_infected, .975),
    .by = elisa_odr
  ) |>
  mutate(
    cuminc_low = cuminc_low,
    cuminc_high = cuminc_high,
    .before = elisa_odr
  ) |>
  kable(
    caption = "Infection probability given an
    ELISA ODR value and a cumulative incidence 95% CI"
  )
```

Table 8: Infection probability given an ELISA ODR value and a cumulative incidence 95% CI

cuminc_low	cuminc_high	elisa_odr	mean	q2.5	q97.5
0.2	0.3	1	0.9422864	0.9203282	0.9591452

Illustration in New-York city and Connecticut

To illustrate this approach, we estimated infection probabilities in New-York City and in Connecticut based on cumulative incidence estimates of (Shioda et al. 2021), using the R code above.

The results are shown in the following table.

Table 9: Infection probability given ELISA ODR in New-York city and Connecticut

location	elisa_odr	Mean (%)	q2.5 (%)	q97.5 (%)
New-York city	0.5	8.4	4.7	13.2
New-York city	0.6	20.7	13.9	27.7
New-York city	0.7	45.6	35.9	54.6
New-York city	0.8	71.7	64.2	77.9
New-York city	0.9	87.6	84.0	90.4
New-York city	1.0	94.8	93.4	96.0
New-York city	1.1	97.8	97.2	98.3
Connecticut	0.5	2.4	1.3	3.9
Connecticut	0.6	6.6	4.0	9.8
Connecticut	0.7	18.6	12.2	25.5
Connecticut	0.8	40.8	30.9	49.9
Connecticut	0.9	65.7	56.8	72.9
Connecticut	1.0	83.1	78.1	87.3
Connecticut	1.1	92.3	89.8	94.3

References

Shioda, Kayoko, Max S. Y. Lau, Alicia N. M. Kraay, Kristin N. Nelson, Aaron J. Siegler, Patrick S. Sullivan, Matthew H. Collins, Joshua S. Weitz, and Benjamin A. Lopman. 2021. "Estimating the Cumulative Incidence of SARS-CoV-2 Infection and the Infection Fatality Ratio in Light of Waning Antibodies." *Epidemiology* 32 (4): 518. https://doi.org/10.1097/EDE.0000000000001361.
