# Supplementary material for: Estimating SARS-CoV-2 infection probabilities with serological data and a Bayesian mixture model
Source: Sci Rep. 2024 Apr 25;14:9503. doi: 10.1038/s41598-024-60060-3 (PMC11045781; doi:10.1038/s41598-024-60060-3)
Supplement: Supplementary file 2 — Supplementary Information 2. [file 41598_2024_60060_MOESM2_ESM.html]

Estimating SARS-CoV-2 infection probabilities with serological data and a Bayesian mixture model


# Estimating SARS-CoV-2 infection probabilities with serological data and a Bayesian mixture model

Supplementary media

Authors

Benjamin Glemain

Xavier de Lamballerie

Marie Zins

Gianluca Severi

Mathilde Touvier

Jean-François Deleuze

SAPRIS-SERO study group

Nathanaël Lapidus

Fabrice Carrat

# Probability of infection in France given age, region and ELISA ODR (optical density ratio)
